# Metastatic Papillary Thyroid Carcinoma with Multifocal Synchronous Transformation to Anaplastic Thyroid Carcinoma

**DOI:** 10.1155/2016/4863405

**Published:** 2016-09-28

**Authors:** Mark Benedict, Jose Costa

**Affiliations:** Department of Pathology, Yale University School of Medicine, New Haven, CT 06510, USA

## Abstract

Papillary thyroid carcinoma is a common malignancy to affect the thyroid and is typified by a nonaggressive nature and low rates of mortality. In contrast, anaplastic thyroid carcinoma is the most aggressive thyroid malignancy with a mortality rate of nearly 90% and survival typically of only six months after the diagnosis is made. The transformation of papillary thyroid carcinoma to anaplastic thyroid carcinoma is well documented in the literature but is uncommon and in most instances is reported as a case report or small series only. Transformation of papillary thyroid carcinoma to anaplastic thyroid carcinoma usually takes place in the thyroid itself or in the adjacent lymph nodes. Only on rare occasions does a transformation occur in a papillary thyroid carcinoma metastasis outside of these locations. In the present case report and subsequent discussion we highlight an unusual case of PTC with transformation to anaplastic thyroid carcinoma, which is shown to involve numerous locations to include near total lung parenchyma obliteration. We also discuss the differential diagnostic challenges when faced with a thyroid malignancy that is negative for thyroglobulin.

## 1. Introduction

Papillary thyroid carcinomas are typically nonaggressive tumors with the survival for stage I disease approximating 100% [1]. These malignancies often show an indolent clinical course, with localized disease commonplace, and typically do not recur or metastasize beyond local lymph nodes [2]. Hence, a simple thyroidectomy is oftentimes curative for these lesions. Carcinoma of the thyroid can occur at any age but peaks earlier in women and is found to be 3 times more common in women when compared to men [1]. Family history of a first-degree relative increases the risk of thyroid carcinoma, although the genetic basis for this is not entirely clear [1]. Certain hereditary conditions are well known to be associated with an increased risk for the development of thyroid cancer and include multiple endocrine neoplasia 2a, multiple endocrine neoplasia 2b, familial adenomatous polyposis, Cowden disease, Carney complex, type I, and familial nonmedullary thyroid carcinoma [1]. Radiation exposure is another well-known risk factor for the development of thyroid carcinoma [1]. In stark contrast to papillary thyroid carcinoma, anaplastic thyroid carcinoma is the most aggressive form of thyroid cancer [3]. Anaplastic thyroid carcinoma comprises less than 5% of total thyroid cancer diagnoses but attesting to its highly aggressive nature boasts a mortality rate of over 90% and mean survival of only six months from the time of its diagnosis [3].

Well-differentiated thyroid carcinomas can dedifferentiate by a multistep process of genetic and epigenetic changes ultimately culminating in a poorly differentiated or undifferentiated/anaplastic carcinoma [2]. The anaplastic transformation of papillary thyroid carcinoma is well-documented occurrence, with the majority of cases transforming in the thyroid gland itself or in the surrounding lymph nodes [4]. However, there are a few case reports/small series of papillary thyroid carcinoma transforming to anaplastic thyroid carcinoma at sites other than the neck, which include the lungs and in one peculiar case the shoulder mimicking a sarcoma [4, 5]. In fact, a study of autopsy cases revealed that the most common sites of distant metastasis in anaplastic thyroid carcinoma include, in decreasing frequency, the lungs (78%), intrathoracic lymph nodes (58%), neck lymph nodes (51%), pleura (29%), adrenal glands (24%), liver (20%), brain (18%), and retroperitoneal lymph nodes (18%) [6]. In the following case report we describe a papillary thyroid carcinoma, which takes on an aggressive form by transforming into a multifocal anaplastic thyroid carcinoma. The burden of disease, distant metastases, and multifocality of transformation in this particular case was incredible and afforded us the opportunity to present an interesting case of transformation.

## 2. Case Report

The patient was a 53-year-old woman who was being worked up for an upcoming hysterectomy when a nodule was noted in the thyroid by a preoperative clearance X-ray. Additionally, several small nodules were noted in the lung, mostly in the lung bases bilaterally. A CT scan was subsequently performed, which confirmed the lung nodules and also showed a 3.4 cm hypoenhancing mass in the left lobe of the thyroid with right sided tracheal displacement. The thyroid mass was biopsied by fine needle aspiration and revealed a papillary thyroid carcinoma (PTC), which was BRAF V600E mutation negative. Approximately 5-6 years prior to her death, she underwent a total thyroidectomy for definitive surgical management as well as radioactive iodine ablation (251 mCi of iodine 131) with no uptake noted in the lung lesions. Surgical pathology at that time revealed a 4.3 cm PTC with oncocytic features, focally severe nuclear atypia, and tumor giant cells. These findings raise the possibility of focal anaplastic transformation at the time of initial resection. Additionally, giant cells have also been reported in cases of anaplastic thyroid transformation [7]. Of note, no extrathyroidal extension was seen at time of resection, margins were negative, and there was no lymph node metastases; however, venous/lymphatic invasion were present. The patient's lung nodules, at that time, were not biopsied and on numerous occasions she was lost to follow-up. Approximately 15 months after thyroidectomy, the patient was noted to have a nonstimulated thyroglobulin level of 134 ng/mL (reference < 0.4). Then three months later a new thyroglobulin level was obtained and was 821 ng/mL (reference < 0.4); however, a radioiodine scan was negative. A PET scan showed findings consistent with intensely metabolically active metastatic disease. This included uptake in the right fossa of Rosenmuller on the left thyroid bed, lymph node masses in the mediastinum, and bilateral hilar and bulky precarinal lymphadenopathy. Numerous intensely avid pleural and parenchymal lung nodules were noted to have increased in size and number compared to the previous CT scan. The lung nodules were biopsied and revealed papillary thyroid carcinoma with psammomatous calcifications and PAX8, TTF-1, and thyroglobulin immunostaining were reported as positive. She also developed an associated malignant pleural effusion, which also was consistent with papillary thyroid carcinoma. The patient was started on sorafenib and 5 months later the medication was changed (to sunitinib) due to progression of disease. Medications were stopped after approximately 10 months due to side effects (significant bone pain). She was admitted to the hospital on numerous occasions after treatment for respiratory symptoms. Her most recent admission occurred 12 days prior to her death for increased shortness of breath and dry cough. During this admission she was found to have a right lower lobe infiltrate and a right pleural effusion with an associated marked increase in her WBC count, which was considered to be of an infectious etiology and was treated with antibiotics. Her thyroglobulin level was noted to be 165 ng/mL (reference < 0.4) at that time. She was transferred to the medical intensive care unit the day before her death due to altered mental status and worsening symptoms where she ultimately died.

## 3. Pathology

**Figure 1:**
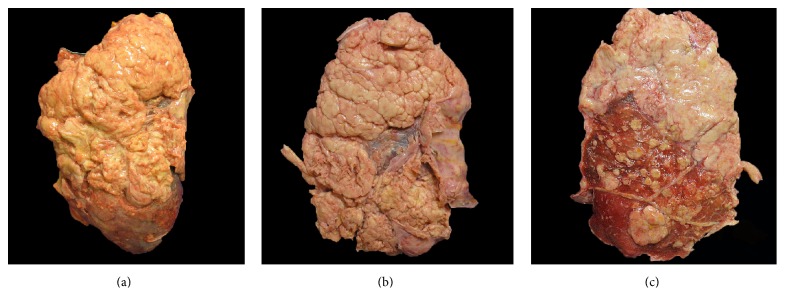
(a) Gross photograph showing the anterior surface of the right lung depicting the extensive involvement by metastatic papillary thyroid carcinoma and transformation to anaplastic thyroid carcinoma (confirmed histologically). (b) Gross photograph showing the left lung with near total encasement by metastatic papillary thyroid carcinoma and anaplastic thyroid carcinoma. (c) Cut surface of the right lung showing extensive tumor involvement with multiple tumoral nodules and complete obliteration of the lung parenchyma (top).

**Figure 2:**
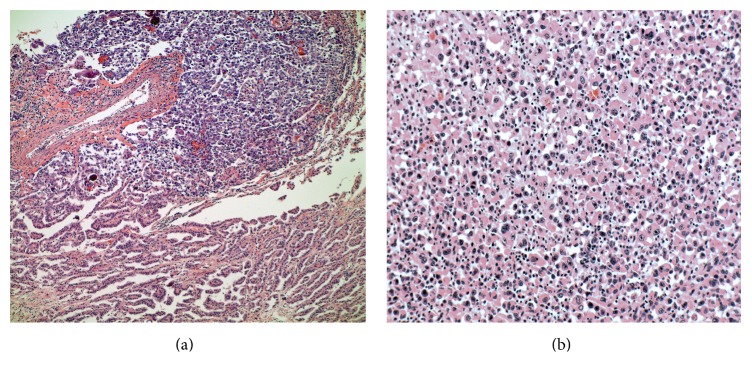
(a) 10x: papillary thyroid carcinoma (lower) with transformation to anaplastic carcinoma (upper). (b) 20x: higher power view of the anaplastic carcinoma component with enlarged nuclei, prominent nucleoli, and moderate amounts of eosinophilic cytoplasm.

**Figure 3:**
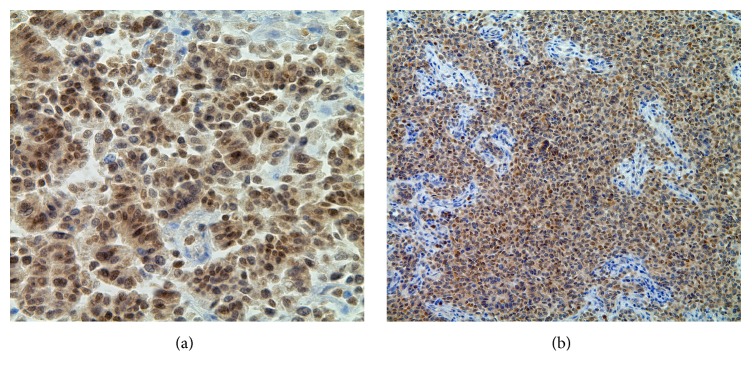
(a) 50x: PAX-8 immunohistochemical stain depicting diffuse nuclear staining in the papillary component of the tumor. (b) 20x: PAX-8 immunohistochemical stain in the solid component of the tumor.

**Figure 4:**
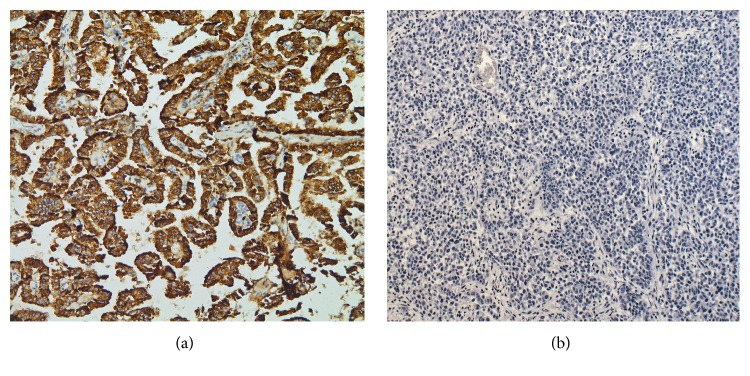
(a) 20x: Napsin-A immunohistochemical stain depicting a focus of diffuse cytoplasmic staining. (b) 20x: thyroglobulin immunohistochemical stain which is negative in the tumor.

**Figure 5:**
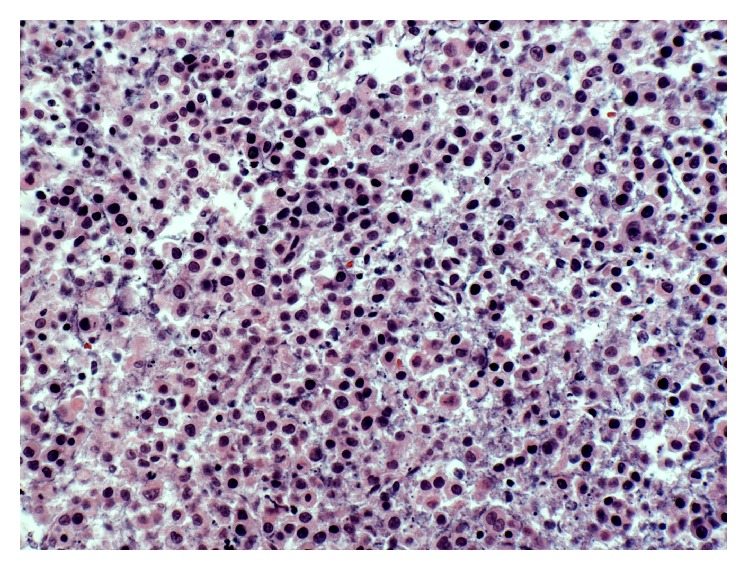
20x: photomicrograph depicting the anaplastic component found to nearly replace the entire right adrenal gland. The cellular morphology here is slightly different from that found in the lung; here we see slightly less eosinophilic cytoplasm and the nucleoli are less prominent.

Metastatic papillary thyroid carcinoma (the bulk majority of tumor present) and coexistent multifocal anaplastic thyroid carcinoma were identified at autopsy involving the bilateral lungs and pleura, pulmonary artery, aorta, pericardium, right adrenal gland, hepatic surface, diaphragm, mediastinal lymph nodes, abdominal lymph nodes, and hilar lymph nodes (Figure 1). Extensive tumor burden was noted in each of these locations; however, the greatest tumor mass was located in the lungs. Microscopic examination revealed diffuse involvement of the lung parenchyma by papillary thyroid carcinoma with adjacent areas of anaplastic transformation (Figure 2). In the well-differentiated areas, the cells are columnar with abundant eosinophilic cytoplasm and focal basal orientation of the nuclei. Nuclear clearing and crowding were noted with nuclear inclusions being inconspicuous. The tumor cells were positive for TTF-1, PAX8 (Figure 3), Napsin-A and were negative for thyroglobulin (Figure 4). The right adrenal gland was essentially replaced by anaplastic tumor, which showed extensive areas of necrosis and a slightly different morphology when compared to the anaplastic component found in the lungs (Figure 5).

## 4. Discussion

While the transformation of papillary thyroid carcinoma to the more aggressive anaplastic carcinoma represents a well-known occurrence, the transformation of metastatic PTC in a distant location (i.e., a location other than the usually seen neck and cervical lymph node metastases) represents an uncommon finding, which typically has been presented in the literature as a case report [8]. Even rarer is the finding of multifocal transformation of PTC to anaplastic carcinoma in distant locations (the lung and adrenal gland in this case) with only a few cases described [4, 9, 10]. The diagnosis of papillary thyroid carcinoma can become challenging, that is, in its differentiation from a primary lung carcinoma, when the thyroglobulin immunohistochemical stain is negative. The immunostains TTF-1, thyroglobulin, PAX8, and Napsin-A are markers that are used in the differential diagnosis of PTC from primary lung adenocarcinoma. In papillary thyroid carcinomas the reactivity of TTF-1, thyroglobulin, and CK7 is essentially 100% [11]. However, that rate drops in poorly differentiated thyroid carcinomas to 86% for TTF-1, 57% for thyroglobulin, and 86% for CK7 [11]. In a study performed by Bejarano et al., one of four anaplastic thyroid carcinomas was focally positive for TTF-1 and all were negative for thyroglobulin and CK7 [11]. The conclusions of Bejarano et al. were that TTF-1 is a more sensitive marker for poorly differentiated thyroid carcinomas and metastases [11]. With that being said, primary lung adenocarcinomas are also positive for TTF-1, which creates diagnostic confusion (if thyroglobulin is negative), when a poorly differentiated or anaplastic thyroid carcinoma is being differentiated from a primary lung adenocarcinoma. Napsin-A is more commonly thought of as being positive in pulmonary adenocarcinomas. However, approximately 5% of all papillary thyroid carcinomas are positive for Napsin-A as well [12]. In addition, it has been shown that 15% of anaplastic, 13% of poorly differentiated, and 100% of micropapillary pattern thyroid carcinomas are positive for Napsin-A [12]. The extreme difficulty in diagnosing metastatic anaplastic (or poorly differentiated for that matter) thyroid carcinoma from a lung adenocarcinoma now becomes clearer. In most cases of anaplastic thyroid carcinoma, thyroglobulin will be negative, while 15% will show Napsin-A positivity. In these instances, PAX-8 can sometimes help in the differential diagnosis of thyroid malignancy versus lung malignancy in that lung adenocarcinomas are typically negative for PAX-8 [12]. PAX-8 has been found to be positive in up 79% of anaplastic thyroid carcinomas [3]. In the case presentation above, the patient's tumor was positive for TTF-1 (could be seen in both thyroid and lung malignancy), Napsin-A (more commonly positive in lung malignancy but also seen in more poorly differentiated thyroid carcinomas), and PAX-8 (more commonly seen in thyroid malignancy), while it was negative for thyroglobulin. Given that the bulk of the patient's tumor was primarily a well-differentiated papillary thyroid carcinoma and the anaplastic component was focal as well as multifocal, it is not surprising that the patient had elevated thyroglobulin levels. The patient's history of having a papillary thyroid carcinoma (noted to have severely atypical nuclear features) coupled with the above immunohistochemistry and the laboratory findings of an extremely elevated thyroglobulin level would be most indicative of a metastatic papillary thyroid carcinoma with anaplastic transformation (multifocal in this case).

The pathogenesis of anaplastic transformation in papillary thyroid carcinoma is still largely unknown. With that being said, advances in molecular technologies have helped by providing some insights into the molecular basis of such a transformation. For example, Xu and Ghossein found the median mutational burden observed in PTC, poorly differentiated thyroid carcinoma, and anaplastic thyroid carcinoma to be 1, 2, and 6, respectively [13]. In fact with the use of whole exome sequencing, the actual median mutational burden in anaplastic thyroid carcinoma was found to be 26 [13]. Results of a recent study on the genomic landscape of poorly differentiated and anaplastic thyroid carcinomas have suggested a stepwise pathogenesis in thyroid malignancy [13]. The investigators proposed a stepwise progression of thyroid carcinoma characterized by increasing mutational burden with greater frequency of mutation seen in the TERT promoter, TP53, EIF1AX, PIK3CA-AKT-mTOR pathway, SWI/SNF complex, mismatch repair genes, and histone methyltransferases [13]. Additionally, molecular evidence points to BRAF and RAS mutations as the main drivers in poorly differentiated and anaplastic thyroid carcinoma [13]. From the molecular evidence above, it is likely that several areas of metastatic PTC foci harbored additional genetic insults leading to anaplastic transformation in these areas.
